# Artificial intelligence-assisted method of structural health monitoring of subway shield tunnel based on insulation degradation location

**DOI:** 10.1371/journal.pone.0325296

**Published:** 2025-06-12

**Authors:** Yiwei Zhao, Chengtao Wang, Yi Tao

**Affiliations:** 1 School of Mechanical, Electronic and Control Engineering, Beijing Jiaotong University, Beijing, China; 2 School of Electrical and Control Engineering, Xuzhou University of Technology, Xuzhou, China; 3 Shanghai Key Laboratory of Rail Infrastructure Durability and System Safety, Shanghai, China; 4 School of Mechatronic Engineering, China University of Mining and Technology, Xuzhou, China; Southwest Petroleum University, CHINA

## Abstract

Stray current leakages inevitably arise to seriously threaten the reliability of the subway tunnel structure and third-party system through electrochemical corrosion. Location of stray current leakage provides novel approach to mitigate stray current corrosion and valid guidance for maintenance of subway tunnel. The present study is dedicated to develop an integrated network-based method for locating the insulation degradation in subway tunnel. In this work, a predictive model based on LWQPSO-SOM algorithm is designed to cluster the risk level of stray current leakage through data mining. Then, an evaluation index *P*_high_ is proposed to calculate the probability of high-risk index in the clustering results. Identification results of leakage zone of stray current is effectively validated through the measurement of rail-to-earth conductance. Results showed that the distribution of *P*_high_ is highly related with rail-to-earth conductance distribution, indicating that the proposed method is applicable for stray current leakage location in the subway tunnel and potentially applicable in engineering fields.

## 1 Introduction

Since firstly operated in 1863, DC rail transit system constitutes the important component of the urban public transportation for its safety, reliability, high capacity, and energy conservation [1–3]. Take Wuhan and Shanghai in China as an example, rail transit shares more than 51% and 70% of public transport traffic respectively [4]. In order to ensure the reliability of reinforced structure in DC mass transit system, multiple protective measures are used to prevent possible accidents generated by hazardous factors such as stray current. ‘Stray current’, also known as the current flowing out of the original flow path, is usually discovered in the urban rail transit system [5–7], causing critical electrochemical corrosion on third-party buried metallic pipelines [8,9] and reinforced concrete structures [10] such as subway tunnel, leading to serious degradation of mechanical properties due to corrosion product accumulation. In the past few years, there have been increasing reports of stray current-induced corrosion and failure of affected underground structures [11,12]. As a typical permanent structure that support the operation of urban rail transit [13], the structural health issue of subway tunnel caused by stray current is corrosion increasingly being concerned by academia and industry.

Stray current is produced due to insulation performance degradation between the running rails and earth, which makes the source of stray current leakage randomly distributed throughout the entire subway line [14–16]. Protective measures have been developed to mitigate stray current attacking on reinforced structure, such as resistance enhancement for rail fastening system [17], rail boot [18], stray current collection mat [19], cathodic protection [20], special design of power and earthing system [21–23], fourth-rail DC railway [24], DC auto-transformer [25], etc. Among all the protective measures, insulation degradation location of rail fasteners could directly provide protective information since rail fasteners provide the only path for stray current leakage. Ground fault detection and location has always been an important issue in power and energy systems, such as DC traction power system [26], low-voltage DC-bus microgrid system [27]. According to the Chinese standard CJJ 49–2020, the area where the value of transition resistance between running rails and structural steel rebar is less than 150 Ω·km is considered to be the area where stray current leakage exists. As the transition resistance decreases, the amplitude of stray current leakage will be decreased correspondingly. The amplitude and direction of stray current leakage is affected by both electrical and environmental factors and operation of trackside equipment (e.g., unidirectional conduction device and over-voltage protection device) [28]. Meanwhile, stray current flows in various elements of subway system, including reinforced rebar in the stray current collection mat and ballast, reinforced rebar in the tunnel lining, metallic facilities, etc. The above two aspects determine that the insulation degradation zone is difficult to accurately describe based on traditional mathematical method. Although the insulation performance of rail fasteners can be described by rail-to-earth conductance, it is time-consuming, inefficient and lack of automation, which is difficult to be applied in engineering. To mitigate stray current leakage, it is critical to maintain the insulation performance of rail fasteners at a high level within the entire subway line. Given this, an artificial intelligence-assisted method is still needed for efficient location of rail-to-earth insulation deterioration zones to provide effective information for monitoring health status of subway tunnel under stray current interference.

As computer technology develops in recent years, intelligent learning technique is emerging with the increasing amount of data in various monitoring systems, which has been employed to develop predictive method to provide critical information for structural health monitoring under stray current interference [29–31]. Through the parameterized finite element model, Liu et al. developed an ANN-based model for predicting failure pressure of high-strength pipes with stray current corrosion defect [32]. Ma et al. proposed a predictive model for corrosion rate of pipeline induced by stray current [33]. The model proved that the negative shift of pipe-to-soil off-potential plays an significant role in the corrosion rate prediction. Wang et al. employed ANN to predict the polarization kinetic parameters in stray current corrosion process and the results demonstrated the feasibility of ANN in stray current corrosion prediction [34]. Li et al. developed a PCA-ELM model to predict amplitude of stray current along the buried gas pipelines based on the field test measurement, which includes pipe-to-soil potential, soil moisture content, soil resistivity, pipeline buried depth, soil pH, amplitude of pipeline current and cathodic protection (CP) current [35]. The amplitude of stray current in [35] was calculated as the absolute value of the subtraction of pipeline current and CP current. Cai et al developed a combined analysis methods of finite element analysis, partial least square and electrochemical measurement technique to predict the interference current of buried pipeline [36]. Wang et al employs DuCOM numerical platform to conduct prediction of ion concentration distribution inside concrete under stray current interference [37]. In the sense of considering the above research, machine learning algorithm models could provide nonlinear mapping relationship between input and output variables, but the studies on their applications in the location of insulation degradation zones haven’t been found. Hence, by applying machine learning algorithm, insulation degradation zones are expected to be identified based on multi-source monitoring data.

**Fig 1:**
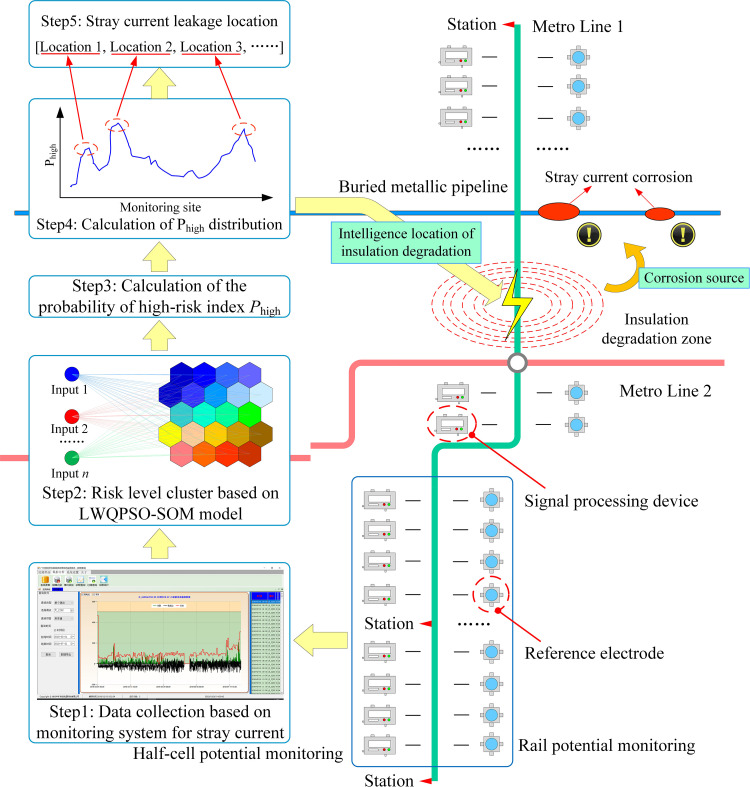
Stray current corrosion on subway shield tunnel induced by insulation degradation of rail fasteners.

Ground fault location is an important task in ensuring the safety and performance of the power supply system [38]. Worldwide scholars have carried out relevant research on this issue in fields such as DC distribution lines [39] and non-directly grounded distribution networks [40]. The possibility of integration with other types of sensors is a feasible measure to improve the performance of the proposed method based on a single sensor, such as ultrasonic technique [41], vibration [42], and fiber grating [43]. However, the location of insulation deterioration during the stray current backflow process still needs further study. The intension of this study is to develop an original approach for intelligent location of insulation degradation of rail fasteners within the entire DC subway line based on the LWQPSO-SOM algorithm and existing sensing information, and therefore to provide critical indicator for health monitoring of reinforced structures of subway tunnel in mainland China, as shown in Fig 1. Self-organizing map (SOM) is employed in this work to conduct the unsupervised clustering of stray current leakage risk at different monitoring sites. And Levy weighted quantum particle swarm optimization (LWQPSO) algorithm is designed to optimize the structural parameters of SOM network to improve the clustering performance. Further, the probability of high-risk index in the clustering results is proposed to finally determine the insulation degradation zone.

Rest of this paper is organized as follows. Section 2 introduces the fundamental theories related to the developed model including SOM and LWQPSO and proposed hybrid methodology. Section 3 presents the prediction procedures comprehensively. Section 4 describes the implementation of the methodology and clustering results of stray current leakage risk based on the monitoring data. Section 5 further discusses the clustering results and assesses the distribution of leakage risk probability of multiple traction sections over the entire subway line. Section 6 finally summarizes the conclusions and future works.

## 2 Methodology

### 2.1 Self-organizing map (SOM)

Self-organizing map is an unsupervised machine learning algorithm that was first introduced by Teuvo Kalevi Kohonen in 1982 [44]. The algorithm is used to explore and visualize patterns between a high-dimensional input space *X* (input layer) and a low-dimensional space *L* (output layer) [45]. Similar samples in input space *X* could be mapped to adjacent neurons in the output layers of the network. Neurons of output layers are arranged in fixed 2-dimensional topological forms and are fully connected to input layer via the weight matrix *W*. Thus, given a classification problem that consists of the set of *N* training vectors *x*_*i*_, the output of the *j*^th^ neuron in output layer *O*_*j*_ is expressed as:


$$O_{j}=\sum_{k=1}^{n}\omega_{jk}x_{k}$$ (1)

where ***x***_*k*_ represents *k*^th^ training vector in the database; *ω*_*jk*_ denotes weight vector between *j*^th^ neuron and input vector ***x***_*k*_.

When conduct training, the distance between the input vector and the weight vector if output neurons are calculated for each output layer neuron. The neuron with the closest distance becomes the best matching unit. The weight vector of the best matching unit and its neighboring neurons are adjusted so that the distance between these weight vectors and input vector is reduced. For *j*^th^ output layer neuron, modification of its weights is calculated as:


$$A_{j}=F_{\min}d_{j}=F_{\min}\left(\sum_{k=1}^{n}{\left(x_{k}-\omega_{jk}\right)}^{2}\right)$$ (2)

$$\Delta \omega_{jk}=A_{j}\eta \left(x_{i}-\omega_{jk}\right)$$ (3)

where *A*_*j*_ is the activation of *j*^th^ output neuron; *F*_min_ represents unity function returning 0 or 1; *d*_*j*_ represents neurons in the neighborhood with *j*^th^ neuron; *η* represents gain term decreasing over time. Different map topologies can be employed in SOM. In this work, classical topology is assumed on a rectangular grid.

### 2.2 Levy weighted quantum particle swarm optimization (LWQPSO)

LWQPSO is an improved quantum particle swarm optimization (QPSO) algorithm that was first proposed by Wang et al in previous studies [46]. The algorithm combines Levy flight search and weighted mean average best position of QPSO to improve optimization performance. For the traditional QPSO algorithm, update equation for particle position [47] can be obtained by:


$$X_{i,j}\left(t+1\right)=p_{i,j}\pm \alpha \left|mbest-X_{i,j}\left(t\right)\right|ln\left(\frac{1}{u_{i,j}\left(t\right)}\right)$$ (4)

$$Rand\left(0,1\right)=\left\{ \begin{array}{c} if:Rand\left(0,1\right)<0.5,+\\ if:Rand\left(0,1\right)>0.5,- \end{array}\right.$$ (5)

where *X*_*i,j*_(*t* + 1) represents *t* + 1^th^ iteration position of the *t*^h^ particle in the *j*^th^ dimension, *X*_*i,j*_(*t*) represents *t*^th^ iteration position of the *i*^th^ particle in the *j*^th^ dimension; *p*_*i,j*_ represents local attractor of the *i*^th^ particle in the *j*^th^ dimension; *α* represents contraction-expansion coefficient; ‘±’ in Eq. (4) is determined according to random function shown in Eq. (5). Given a population with *M* particles of *D* dimensions, *mbest* in Eq. (4) represents weighted mean average best position that is calculated as:


$$mbest=\frac{1}{M}\sum_{i=1}^{M}pbest=\left(\frac{1}{M}\alpha_{i,1}\sum_{i=1}^{M}pbest_{i,1},\frac{1}{M}\alpha_{i,2}\sum_{i=1}^{M}pbest_{i,2},...,\frac{1}{M}\alpha_{i,D}\sum_{i=1}^{M}pbest_{i,D}\right)$$ (6)

where *pbest* represents; [*α*_*i*,1_, *α*_*i*,2_, …, *α*_*i*,*D*_] is a series of weight coefficients with certain regularity.

Levy flight search is used in this model to update the global best position for each iteration. Mantegna method is used to simulate the Levy distribution [48]. Hence, global best position after jump mutation can be expressed as:


$$G{\left(t\right)}^{\prime }=G\left(t\right)+\alpha ⊕\frac{\mu }{{\left|\upsilon \right|}^{\frac{1}{\lambda -1}}}$$ (7)

where *μ* ~ *N*(0, *σ*_*μ*_^2^), *υ* ~ *N*(0, *σ*_*υ*_^2^); *G*(*t*)’ denotes global best position after Levy mutation; *G*(*t*) denotes global best position. Variance *σ*_*μ*_^2^ and *σ*_*υ*_^2^ can be obtained through Eq. (8).


$$\left\{ \begin{array}{c} \sigma_{\mu }=[\frac{\Gamma (1+\chi )\cdot sin\left(\frac{\chi }{2}\pi \right)}{\Gamma \left(\frac{1+\varphi }{2}\right)\cdot \chi \cdot 2^{\frac{\varphi -1}{2}}}\backslash \sigma_{\upsilon }=1 \end{array}\right.$$ (8)

### 2.3 Proposed hybrid model LWQPSO-SOM

The model proposed in this work ensembles SOM and LWQPSO. The structure of SOM will directly affect cluster accuracy and stability. The performance of SOM is highly dependent on structural parameters, but these parameters usually require manual experience adjustment and are sensitive to data distribution. In this work, the use of LWQPSO is to improve the prediction performance of SOM network by avoiding falling into local optimum. In order to further improve the performance of classification, LWQPSO algorithm is employed in this work to optimize the structural parameters of output layer. Fitness function is defined as Eq. (9) to measure whether the termination requirement is met. In the fitness function, Davies-Bouldin Index (DBI) is to measure the accuracy of classification [49], where $\overset{―}{S_{n}}$ denotes the average Euclidean distance between the n^th^ sample and its center, *w*_*n*_ denotes the center of *n*^th^ sample. The smaller DBI indicates smaller intra-class distance and larger inter-class distance. The proposed hybrid model’s pseudocode is shown in Algorithm 1.


$$DBI=\frac{1}{N}\sum_{n=1}^{N}\underset{m\neq n}{\max}\left(\frac{\overset{―}{S_{n}}+\overset{―}{S_{m}}}{{\left\|w_{n}-w_{m}\right\|}_{2}}\right)$$ (9)

Algorithm 1. LWQPSO-SOM

Input: Initialize training dataset *S*_train_ and datatest set *S*_test_

Output: Clustering results

Initialize the particle optimization and weights of SOM network

While (*n* < *G*_max_)

Calculate linear descent inertia weight and average best position

for each particle *j* (1 ≤ *j* ≤ *M*) in swarm do

Encode weight vector based on the initialization of SOM

Train *S*_train_ using SOM

Calculate the fitness of the particle according to Eq. (9)

 If the fitness of the particle < the fitness of pbest then

  pbest is set as *P*_*i,j*_(*t*)=*X*_*i,j*_(*t*)

 End if

End for

for each individual best position *P*_*i*_(*t*) (1 ≤ *i* ≤ *N*) do

 Determine the global best position *G*(*t*) through *G*(*t*)=*P*_*g*_(*t*), *g* = argmin1 ≤ *i ≤ N f*{*f*[*P*_*i,j*_(*t*)]}

End for

for each particle *j* (1 ≤ *j* ≤ *M*) in swarm do

 Calculate the linear descent inertia weight

 Calculate the average best position according to Eq. (6)

 Update the position of the particle according to Eq. (4)

End for

Store the fitness for each generation;

Update the position of global optimal particle according to Eq. (7)

Until the preset termination condition is met

Set the structure of SOM based on the global best position *G*(*t*)

Test the trained SOM through *S*_test_

Used the trained SOM to cluster the level of stray current leakage *R*

## 3 Prediction procedures

**Fig 2:**
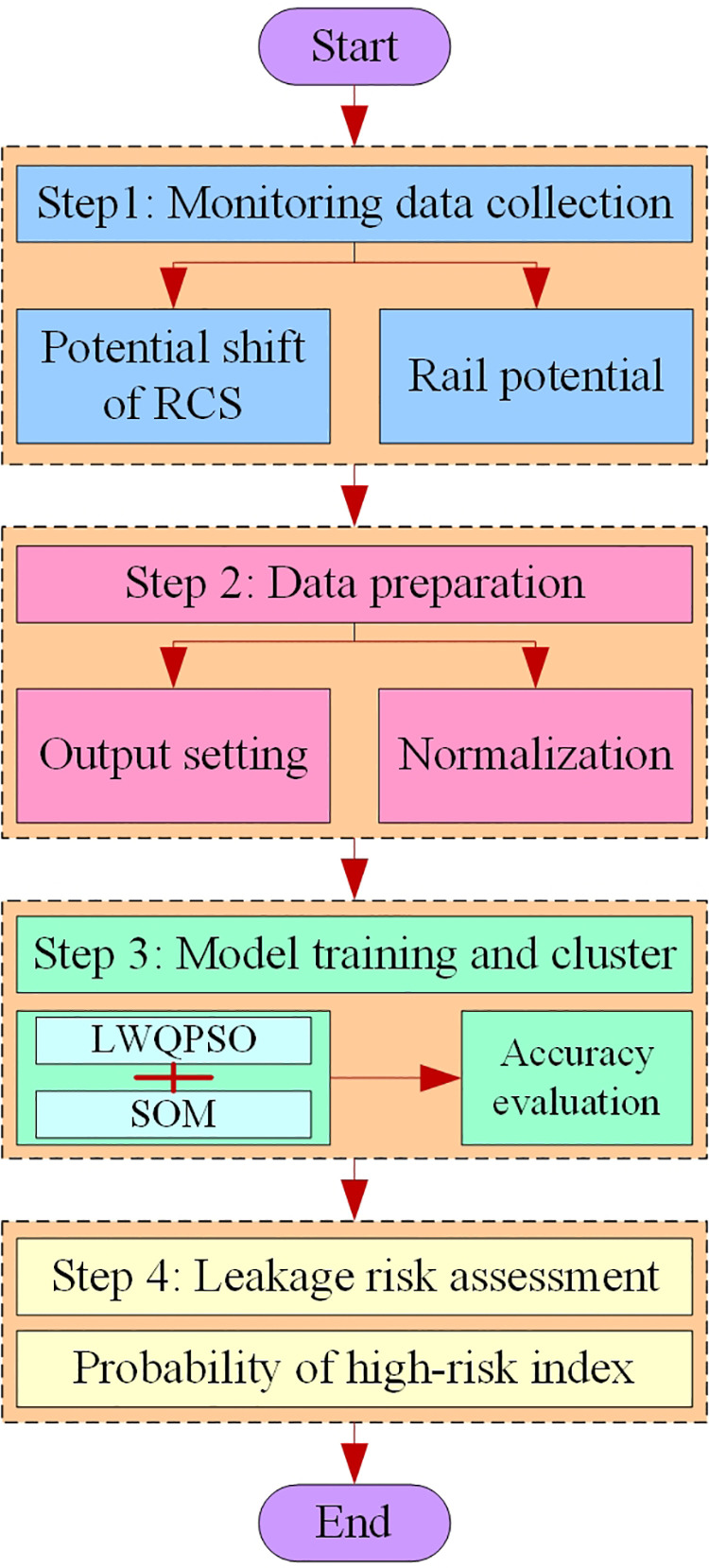
Location of insulation deterioration zone in shield tunnel of DC rail transit system based on LWQPSO-SOM network.

Fig 2 shows the flowchart of the proposed approach for stray current leakage localization. The main procedures include:

- Sensing data collection
- Data preparation
- Model training and cluster
- Leakage risk assessment

### 3.1 Data collection based on monitoring system

**Fig 3:**
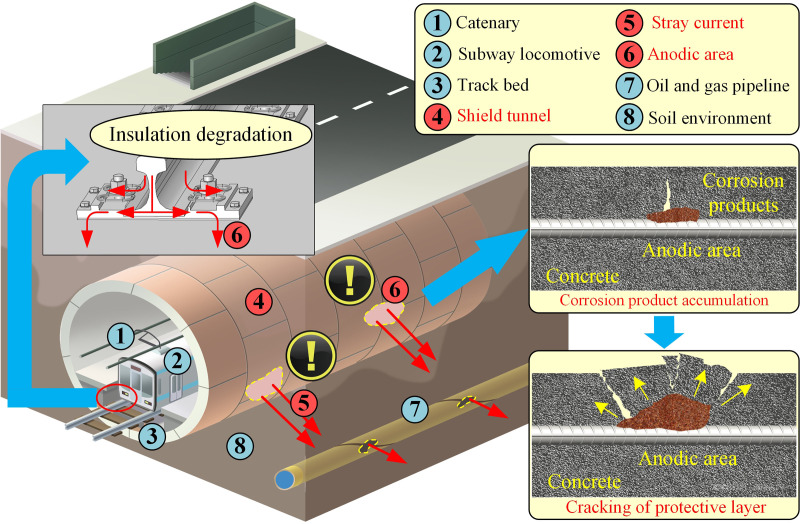
Flowchart of the stray current leakage localization.

**Fig 4:**
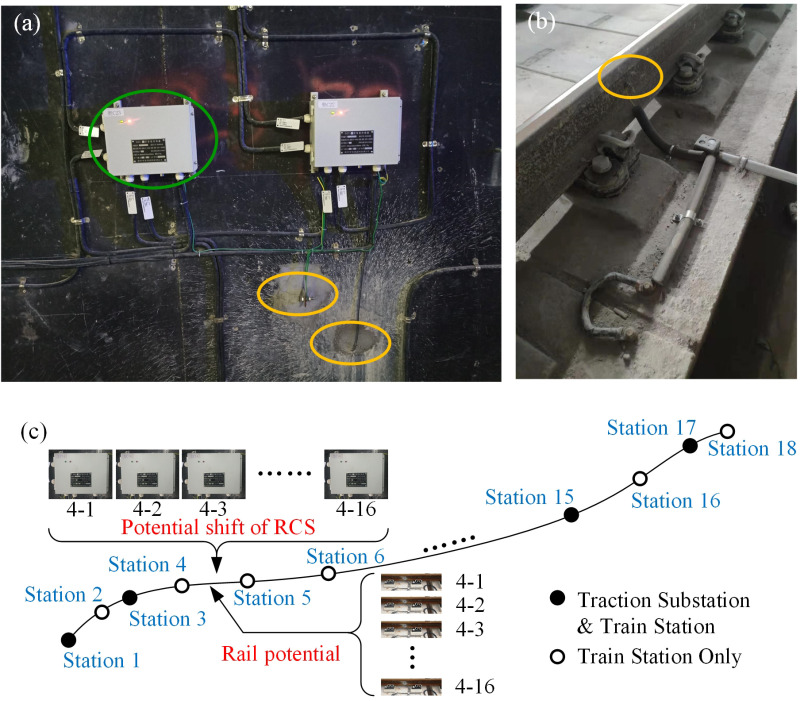
Data collection in the DC rail mass transit system. (a) Potential shift of RCS. (b) Rail potential. (c) Sensor distribution in the subway line.

Aforementioned in Section 1, stray current leakage from subway system generates serious electrochemical corrosion on both shield tunnel structure and buried metallic pipelines, as shown in Fig 3. Hence, this study employs the data related to stray current from the subway system itself to achieve accurate location task. Data employed in this study were collected from the monitoring system of rail potential and half-cell potential of shield tunnel, which has been existed as a part of the safety monitoring system since the metro construction. Sensors of rail potential and reference electrodes are evenly distributed in each power section over the entire subway line, as shown in Fig 4. The monitoring system is composed of reference electrodes, microprocessor for half-cell potential signal, adapter, host computer, and communication cable. The monitoring system realizes multi-point monitoring of half-cell potential through a LAN-based networking method. The microprocessor performs digital signal conversion and employs fieldbus for communication, and can also be used as a transmission signal line junction box. The reference electrode used is Mo/MoO_3_ electrode for the metro line shown in Fig 4. The position of yellow ellipse in Fig 4 denotes the installation location of reference electrode and rail potential measurement. There are 242 rail potential sensors connected to track side and 242 reference electrodes pre-embedded in the sidewall of the tunnel. The installation positions of rail potential sensors and references is the same along the coordinate in the direction of locomotive running. Therefore, a total of 242 monitoring sites are set within 17 power supply sections. Measured signals of potential shift of RCS and rail potential were firstly transmitted to the intelligent device (green ellipse) that shown in Fig 4a for preliminary processing and then uploaded to industrial personal computer in the integrated monitoring center. All the data was collected from Nov. 2021 to Feb. 2022. Exemplary collected data are listed in Table 1. The number of sensors equipped within different power supply sections are determined according to the length of interval. The distance between adjacent sensors is fixed. Therefore, different numbers of sensors are installed in different lengths of power supply sections. Parameters presented in Table 1 includes natural potential of reinforced concrete structure (RCS) *E*_*n*_, maximum potential shift of RCS Δ*E*_max_, minimum potential shift of RCS Δ*E*_min_, mean positive potential shift of RCS $\overset{―}{\Delta E_{+}}$, mean negative potential shift of RCS $\overset{―}{\Delta E_{-}}$, maximum rail potential of RCS *P*_max_, minimum rail potential of RCS *P*_min_, mean positive rail potential of RCS $\overset{―}{P_{+}}$, mean negative rail potential of RCS $\overset{―}{P_{-}}$. There are 17 power supply sections along the entire subway line, as given in Fig 4c. The above parameters were calculated for every 0.5 h according to the collected rail potential signal and half-cell potential signal. Sensor No. indicates the specific location of the monitoring sensor. Take ‘3 - 2’ in Table 1 as an example, index ‘3’ represents 3^rd^ power supply section and index ‘2’ represents 2^nd^ distributed monitoring sensor.

**Table 1:** Exemplary monitoring data for the location of stray current leakage area.

No.	Sensor Label	*E*_*n*_ (mV)	Δ*E*_max_ (mV)	Δ*E*_min_ (mV)	$\overset{―}{\Delta E_{+}}$ (mV)	$\overset{―}{\Delta E_{-}}$ (mV)	*P*_max_ (V)	*P*_min_ (V)	$\overset{―}{P_{+}}$ (V)	$\overset{―}{P_{-}}$ (V)	Monitoring time
1	1 − 1	145	239	35	10	0	25	19.6	5	0	2022-02-01 00:00:00
2	1 - 2	2	401	138	52	0	26	0	3	0	2022-02-01 00:00:00
3	1 - 3	69	13	2	3	0	7	0	5	0	2022-02-01 00:00:00
4	1 - 4	141	0	−251	0	−55	29	10.6	3	0	2022-02-01 00:00:00
5	1 - 5	183	27	−255	13	−67	53	6.6	9	0	2022-02-01 00:00:00
6	1 - 6	168	−3	−249	0	−51	64	4.6	10	0	2022-02-01 00:00:00
7	1 - 7	231	0	−246	0	−47	45	−32	27	−19.3	2022-02-01 00:00:00
8	1 - 8	76	23	−71	17	−13	18	−17	4	−9.6	2022-02-01 00:00:00
9	1 - 9	70	48	−195	41	−32	16	0	9	0	2022-02-01 00:00:00
10	1 - 10	97	7	−250	2	−160	61	0	31	0	2022-02-01 00:00:00
…	…	…	…	…	…	…	…	…	…	…	…
121742	2 − 1	105	29	0	33	0	104	11.5	42	0	2022-02-11 15:00:00
121743	2 − 2	102	93	−199	54	−181	115	20.6	45	0	2022-02-11 15:00:00
…	…	…	…	…	…	…	…	…	…	…	…
181772	3 − 1	42	31	2	6	0	59	−22	12	−14.6	2022-02-16 20:30:00
181773	3 − 2	48	19	−26	8	−13	57	0	19	0	2022-02-16 20:30:00
…	…	…	…	…	…	…	…	…	…	…	…
316052	17 −14	272	188	10	140	0	50	1.6	16	0	2022-02-28 14:00:00

### 3.2 Database establishment and preparation

The purpose of this section is to conduct preliminary processing of the sensing data shown in Section 3.1, which includes database construction, data normalization and sample separation.

(1) In the established prediction model, the input variables are the natural potential *E*_*n*_, maximum potential shift Δ*E*_max_, minimum potential shift Δ*E*_min_, mean positive potential shift $\overset{―}{\Delta E_{+}}$, mean negative potential shift $\overset{―}{\Delta E_{-}}$, maximum rail potential *P*_max_, minimum rail potential *P*_min_, mean positive rail potential $\overset{―}{P_{+}}$, mean negative rail potential $\overset{―}{P_{-}}$. The output variable is the risk level of stray current leakage *R*. Risk of stray current leakage is divided into different levels. For example, level index *R*_1_, *R*_2_, *R*_3_, *R*_4_ and *R*_5_ are implemented by outputting vectors *R*_1_ = [1, 0, 0, 0, 0], *R*_2_ = [0, 1, 0, 0, 0], *R*_3_ = [0, 0, 1, 0, 0], *R*_4_ = [0, 0, 0, 1, 0] and *R*_5_ = [0, 0, 0, 0, 1]. In this work, 15 indexes are employed to cluster the risk level of stray current leakage, as given in Table 2. Hence, on accounting of the calculated probability of stray current risk and hazard evaluation criterion proposed by Zakowski and Sokolski [50]. Hence, it is proposed in this paper that high risk of stray current leakage is identified from index 8 (*R*_11_ = [0, 0, 0, 0, 0, 0, 0, 1, 0, 0, 0, 0, 0, 0, 0]) to index 15 (*R*_15_ = [0, 0, 0, 0, 0, 0, 0, 0, 0, 0, 0, 0, 0, 0, 1]).
(2) Normalization process is required for preprocessed data shown in Table 1 to limit to a certain range (e.g., [0, 1] in this work). After data normalization, the speed of gradient descent to find the optimal solution is improved. Min-max normalization is performed in this paper according to Eq. (10).

**Table 2:** Division rules for clustering risk index of stray current leakage.

Risk index	Network output
Index 1	[1, 0, 0, 0, 0, 0, 0, 0, 0, 0, 0, 0, 0, 0, 0]
Index 2	[0, 1, 0, 0, 0, 0, 0, 0, 0, 0, 0, 0, 0, 0, 0]
Index 3	[0, 0, 1, 0, 0, 0, 0, 0, 0, 0, 0, 0, 0, 0, 0]
Index 4	[0, 0, 0, 1, 0, 0, 0, 0, 0, 0, 0, 0, 0, 0, 0]
Index 5	[0, 0, 0, 0, 1, 0, 0, 0, 0, 0, 0, 0, 0, 0, 0]
Index 6	[0, 0, 0, 0, 0, 1, 0, 0, 0, 0, 0, 0, 0, 0, 0]
Index 7	[0, 0, 0, 0, 0, 0, 1, 0, 0, 0, 0, 0, 0, 0, 0]
Index 8	[0, 0, 0, 0, 0, 0, 0, 1, 0, 0, 0, 0, 0, 0, 0]
Index 9	[0, 0, 0, 0, 0, 0, 0, 0, 1, 0, 0, 0, 0, 0, 0]
Index 10	[0, 0, 0, 0, 0, 0, 0, 0, 0, 1, 0, 0, 0, 0, 0]
Index 11	[0, 0, 0, 0, 0, 0, 0, 0, 0, 0, 1, 0, 0, 0, 0]
Index 12	[0, 0, 0, 0, 0, 0, 0, 0, 0, 0, 0, 1, 0, 0, 0]
Index 13	[0, 0, 0, 0, 0, 0, 0, 0, 0, 0, 0, 0, 1, 0, 0]
Index 14	[0, 0, 0, 0, 0, 0, 0, 0, 0, 0, 0, 0, 0, 1, 0]
Index 15	[0, 0, 0, 0, 0, 0, 0, 0, 0, 0, 0, 0, 0, 0, 1]


$$x_{i}^{\prime }=\frac{x_{i}-x_{\min}}{x_{\max}-x_{\min}}$$ (10)

where *x*_*i*_*’* represents *i*^th^ normalized data; *x*_*i*_ represents *i*^th^ raw data; *x*_max_ represents maximum raw data; *x*_min_ represents minimum raw data.

(3) The division of database is vital to model training and testing. Before inputing the normalized data into the clustering model, database in Section 3.1 is randomly divided for training set and test set with a percentage of 70% and 30%, respectively. Total number of sample set is 310652. Theoretically, the number of training set and test set is 217456 and 93196 respectively. Random sampling of the training samples and test samples is adopted to reduce the time required for algorithm training.

### 3.3 Model training and cluster

In this work, the training and optimization of the proposed are conducted simultaneously for obtaining final optimized network structure. When LWQPSO finds the best structural parameters of the SOM, the training of SOM also terminates. It should be mentioned that in the proposed method each weight in the vector *ω*_*jk*_ was firstly set in the range of [−1, 1]. Basic parameters of LQPSO-SOM are listed in Table 3. Besides, convergence condition of the optimization process is set as the maximum number of iterations. Indicators for evaluating the performance of the algorithm are compactness (CP), DBI, Dunn Validity Index (DVI), Calinski-Harabasz Index (CHI), and Silhouette Coefficient (SC) and their mathematical expressions are given by Eqs. (9, 11–14), where Ω_*m*_ represents *m*^th^ cluster, *x*_*i*_ represents *i*^th^ sample in the *m*^th^ cluster Ω_*m*_, *Q* represents the number of clustering sample, *D*_*i*_ represents the average distance between the i^th^ sample and other samples in the same cluster, *D*_*i*_^’^ represents the average distance between the i^th^ sample and samples in other clusters except for Ω_*m*_ (*x*_*i*_ ∈ Ω_*m*_). It should be noted that, with better cluster performance, CP and SP are smaller, while DVI, CHI and SC are bigger.

**Table 3:** Parameters of LWQPSO-SOM algorithm.

LWQPSO		SOM	
Population size *M*	10	Topology function	*hextop*
Maximum generation *G*_max_	50	Distance function	*Linkdist*
Initial value of C-E coefficient	0.7	Steps for neighborhood to shrink to 1	100
Final value of C-E coefficient	0.2	Initial neighborhood size	3
Constant *λ*	1.5	Epoch	10
Initial value of linear drop weights	1.5		
Final value of linear drop weights	0.5		


$$\overset{―}{CP}=\frac{1}{N}\sum_{n=1}^{N}\overset{―}{CP_{n}}\;\overset{―}{CP_{n}}=\frac{1}{\left|\Omega_{n}\right|}\sum_{x_{i}\in \Omega_{n}}\left\|x_{i}-w_{n}\right\|$$ (11)

$$DVI=\frac{\underset{0<m\neq n<N}{min}\left[\underset{\forall x_{i}\in \Omega_{m},\forall x_{j}\in \Omega_{n}}{min}\left(\left\|x_{i}-x_{j}\right\|\right)\right]}{\underset{0<m\leq N}{max}\left[\underset{\forall x_{i},x_{j}\in \Omega_{n}}{max}\left(\left\|x_{i}-x_{j}\right\|\right)\right]}$$ (12)

$$CHI=\frac{tr\left(\sum_{n=1,m\neq n}^{N}S_{n}\left(w_{n}-w_{m}\right){\left(w_{n}-w_{m}\right)}^{T}\right)\left(Q-N\right)}{tr\left(\sum_{n=1}^{N}\sum_{x_{i}\in \Omega_{n}}S_{n}\left(x_{i}-w_{n}\right){\left(x_{i}-w_{n}\right)}^{T}\right)\left(N-1\right)}$$ (13)

$$SC_{i}=\left\{ \begin{array}{c} 1-\frac{D_{i}}{D_{i}^{\prime }},ifD_{i}<D_{i}^{\prime }\\ 0,ifD_{i}=D_{i}^{\prime }\\ \frac{D_{i}^{\prime }}{D_{i}}-1,ifD_{i}>D_{i}^{\prime } \end{array}\right.\;SC=\frac{1}{Q}\sum_{i=1}^{Q}SC_{i}$$ (14)

### 3.4 Leakage location based on stray current risk assessment

Due to the dynamic characteristics of stray current distribution, the same monitoring site cannot be identified as low-risk index for the entire monitoring time. For instance, the same monitoring site may be recognized as high-risk index within a period of time, while recognized as low-risk index within the remaining time. Within 24 h monitoring period, cluster task will be carried out on each monitoring site for 767 times. For the same monitoring site, its clustering result may be identified low-risk index at one time, and then identified as high-risk index at another time. This phenomenon is related to variation in humidity caused by seasonal climate change and daily maintenance to reduce oil pollution and iron filings. Therefore, based on the clustering results of risk level of stray current leakage, the probability of high-risk index of the same monitoring site *P*_high_ is calculated through Eq. (15) for locating insulation degradation zones within a certain period of time (e.g., one month in this work), where *N*_high_ denotes the number of sample data identified as high-risk index under stray current leakage for the same monitoring site, *N*_total_ denotes total number of sample data for the same monitoring site, and *N*_total _= 767 for one month. If the value of *P*_high_ is calculated to be large, it indicates that the nearby zone where the sensor is located is prone to leaking stray current during most operation time. Only the monitoring site that maintains a high probability of being clustered as high-risk index with a certain period will be recognized as insulation degradation zone.


$$P_{high}=\frac{N_{high}}{N_{total}}$$ (15)

## 4 Results and discussion

### 4.1 Clustering results

**Fig 5:**
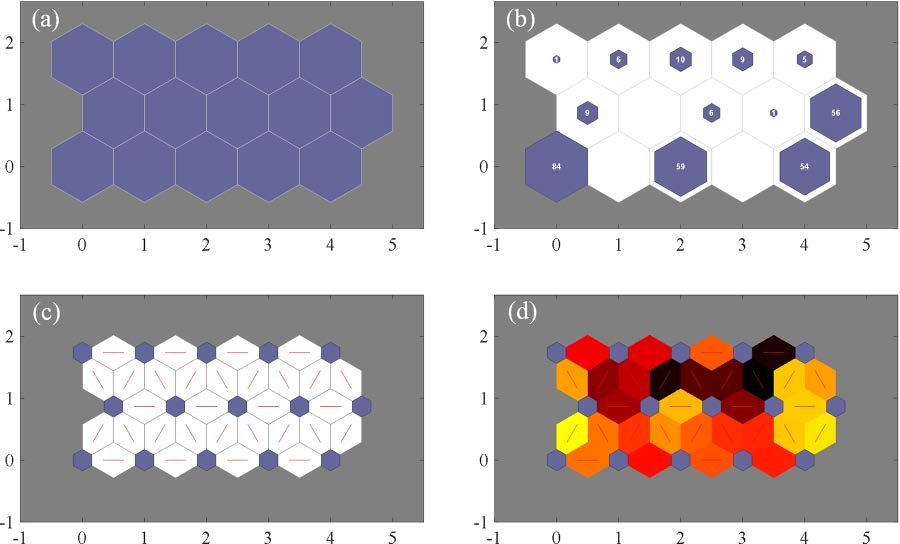
SOM model integrated with LWQPSO optimization. (a) Output structure. (b) Testing results. (c) Weight connection. (d) Neighboring weight distances.

**Fig 6:**
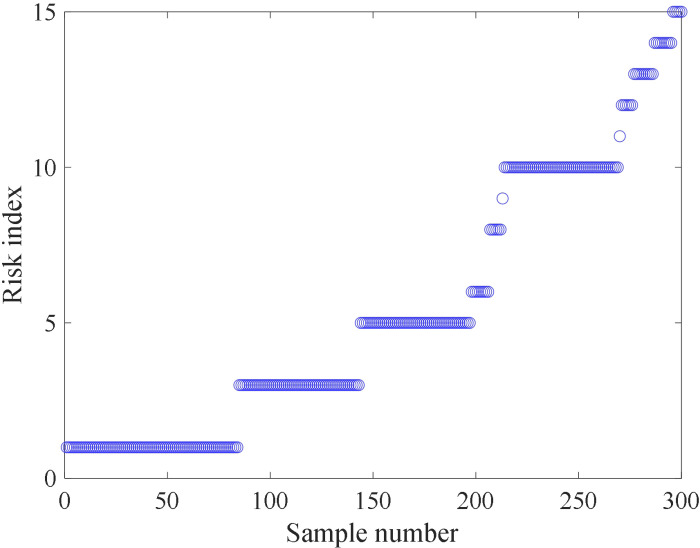
Clustering results of LWQPSO-SOM algorithm.

After the structural optimization based on LWQPSO algorithm, topology of the established unsupervised SOM model is presented in Fig 5 with U-matrix representation. It is seen that the SOM network is optimized as a 5 × 3 output layer and totally 15 indexes are classified with respect to the risk level of stray current leakage from low-risk to high-risk through improved self-organizing data mining. The number on each node in Fig 5b means the number corresponding to each risk level in the test sample. It is observed that different risk levels are successfully divided by the SOM network, which also confirmed that the constructed dataset covers basic measurement data for each risk level. Detailed distribution of clustering results is given in Fig 6. In Fig 5d, yellow node means short distance between 2 nodes in the trained SOM, and red or brown color means longer distance between nodes. Each hexagonal node of the map is associated with a level index related to the risk of stray current leakage. According to the index definition in Section 3.2, clustering results based on the LWQPSO-SOM algorithm is shown in Fig 6 by finding the closest reference vector to each set of the test sample. In 300 test samples that randomly selected from database, 36 samples are identified as high-risk index.

### 4.2 Comparison of different algorithms

**Fig 7:**
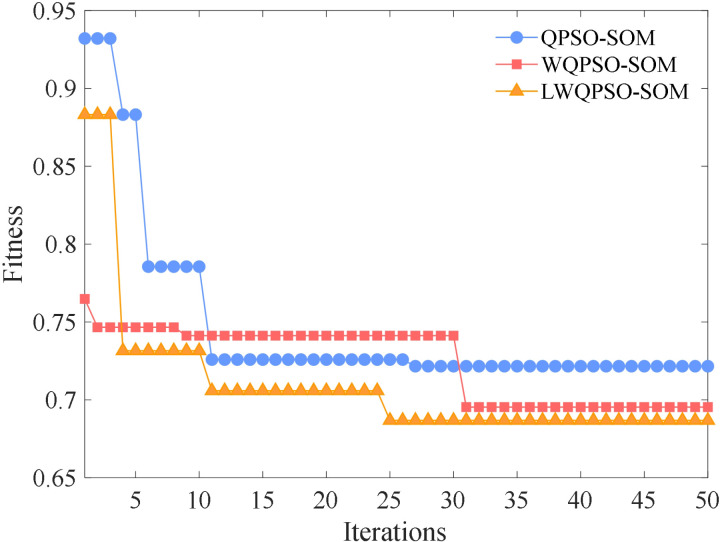
Fitness versus iterations based on different clustering algorithms.

**Fig 8:**
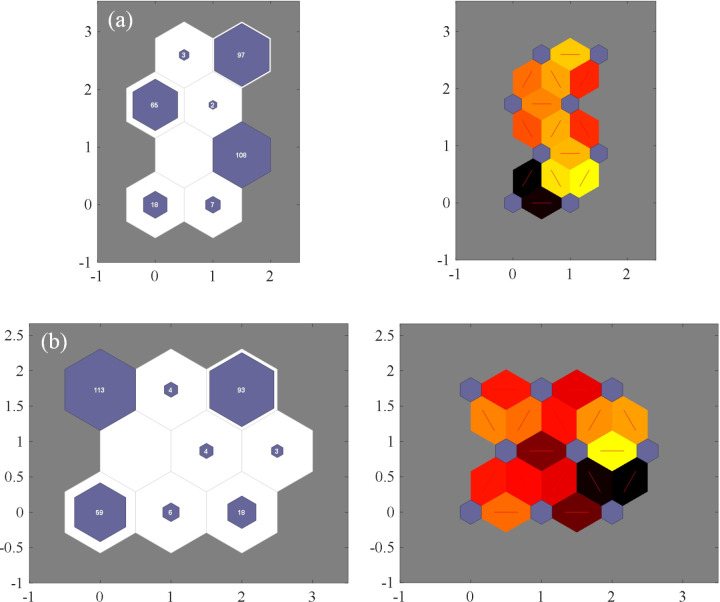
Clustering results and neighboring weight distance of different models. (a) QPSO-SOM. (b) WQPSO-SOM.

**Fig 9:**
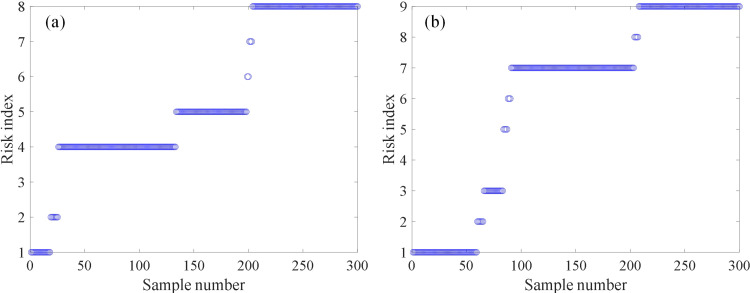
Clustering results of different models. (a) QPSO-SOM. (b) WQPSO-SOM.

To further validate the effectiveness of the proposed LWQPSO-SOM algorithm in the clustering task, QPSO-SOM and WQPSO-SOM are also applied for clustering stray current risk. It is also defined in QPSO-SOM and WQPSO-SOM model that the top 50% of the clustering level is considered as the high-risk index of stray current leakage. Fitness value versus the number of iterations based on QPSO-SOM, WQPSO-SOM and LWQPSO-SOM algorithm is shown in Fig 7. The final fitness value of QPSO-SOM, WQPSO-SOM and LWQPSO-SOM is 0.7216, 0.6954 and 0.6868, respectively. There’s no phenomenon of local optimum in the LWQPSO-SOM algorithm. It is noted that LWQPSO-SOM algorithm exhibits the fastest convergence speed and the best convergence accuracy compared with QPSO-SOM and WQPSO-SOM. Clustering results and neighboring weight distances of QPSO-SOM and WQPSO-SOM model are shown in Fig 8 with 2-dimensional visualization. Clustering results along with the risk index of QPSO-SOM and WQPSO-SOM is shown in Fig 9. It is observed that 8 and 9 levels are classified for stray current risk according to the results carried out by QPSO-SOM and WQPSO-SOM model. Meanwhile, it is observed from Fig 9 that, compared with LWQPSO-SOM, more sensing data is identified as high-risk index through the QPSO-SOM and WQPSO-SOM model. Indicators for evaluating unsupervised cluster performance are listed in Table 4. It is seen that, no matter in terms of CP, DBI, DVI, CHI or SC, the proposed LWQPSO-SOM exhibits superiority in classification task compared with QPSO-SOM and WQPSO-SOM.

**Table 4:** Performance indicators of QPSO-SOM, WQPSO-SOM, and LWQPSO-SOM model.

Clustering algorithm	QPSO-SOM	WQPSO-SOM	LWQPSO-SOM
CP	0.8306	0.2946	0.0553
	0.5576	0.2574	0.2946
	0.2089	0.8306	0.2126
	0.3706	0.4075	0.1032
	0.0165	1.0114	0.2574
	0.6114	0.2547	0.1348
	0.1321	0.4535	0.1138
		0.0752	0.2309
			0.8560
			0.5665
			0.7413
			0.1234
DBI	0.7216	0.6954	0.6899
DVI	0.2881	0.3676	0.4107
CHI	228.9208	220.7114	217.1031
SC	0.8627	0.8209	0.7961

**Fig 10:**
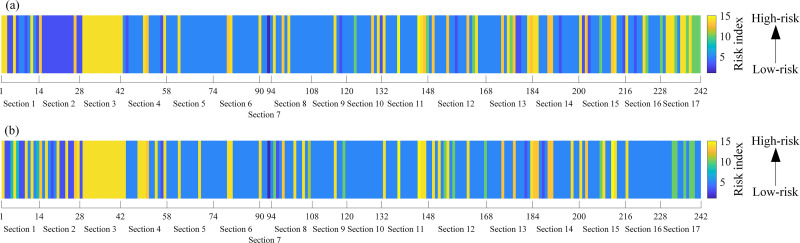
Risk distribution of stray current leakage evaluated along the entire subway line at 2022-02-15. (a) 8:00:00 to 8:30:00. (b) 18:00:00 to 18:30:00.

**Fig 11:**
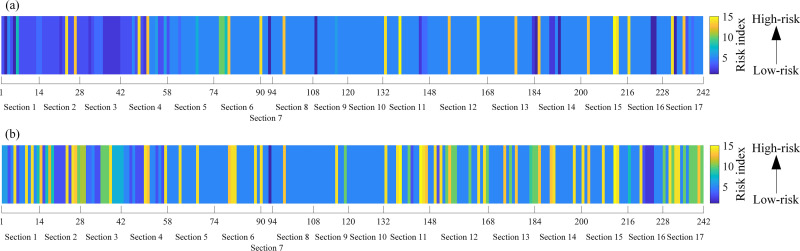
Risk distribution of stray current leakage evaluated along the entire subway line at 2022-02-15 over the entire subway line. (a) 4:00:00 to 4:30:00. (b) 15:00:00 to 15:30:00.

On account of the established QPSO-SOM, WQPSO-SOM, and LWQPSO-SOM model, collected sensing data of the entire subway line from 2022-02-15 8:00:00–8:30:00 and 18:00:00–18:30:00 (traffic peak in the morning and afternoon) was used to conduct the classification considering that test samples are randomly selected, and results are shown in Fig 10. It can be noted from Fig 10 that high-risk insulation degradation zones are identified from 2022-02-15 8:00:00–8:30:00 and 18:00:00–18:30:00., especially for section 3 (power supply section between Station 3 and Station 4). Besides, distribution of risk index is compared between operating hours and stop hours. Take sensing data collected from 2022-02-15 4:00:00–4:30:00 and 15:00:00–15:30:00 as an example, clustering results are shown in Fig 11. It can be noted from Fig 11 that, since the locomotive operation stops at night, risk of stray current leakage generally remains at low-risk index. The phenomenon that there still exists some certain monitoring sites with high-risk index may result from measurement error and synergistic impact of other ground metallic structures.

### 4.3 Leakage risk assessment

**Fig 12:**
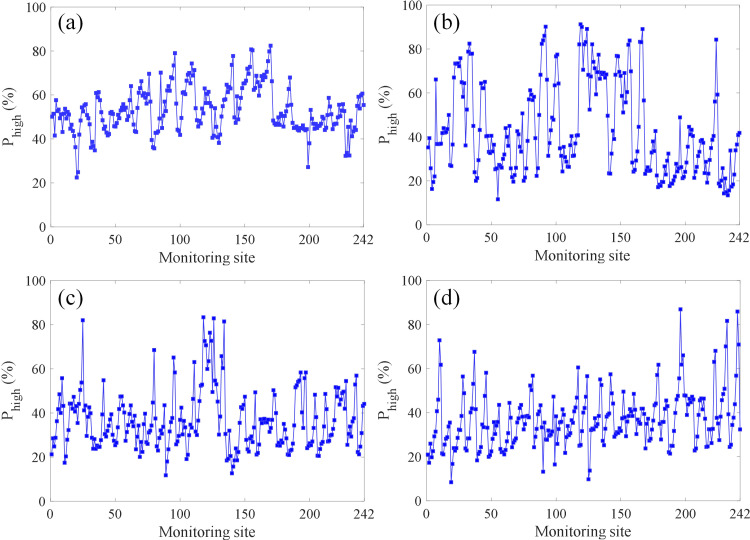
Distribution of Phigh along the entire subway line. (a) Nov. 2021. (b) Dec. 2021. (c) Jan. 2022. (d) Feb. 2022.

Based on the proposed LWQPSO-SOM model in this work, the probability of high-risk index *P*_high_ is evaluated over the entire subway line according to the sensing data collected within four months from Nov. 2021 to Feb. 2022 and Eq. (14), which is shown in Fig 12. It is seen from Fig 12 that the insulation degradation zone is identified based on the proposed method, which is the monitoring site corresponding to the probability of high-risk index. The highest probability *P*_high_ is 82.40%, 91.22%, 83.37% and 86.88% for Nov. 2021, Dec. 2021, Jan. 2022, and Feb. 2022, respectively. And their average value is 53.07%, 43.36%, 36.82% and 36.77%. It could also be seen from Fig 12 that, compared with Dec. 2021, Jan. 2022 and Feb. 2022, distribution of *P*_high_ in Nov. 2021 is relatively concentrated, which means that insulation performance of the entire subway line remains stable during this time. For instance, high-risk index exists in the interval 2, 3, 6, 7, 9, 10, 11 and 16 during Dec. 2021. This means that, in these power supply sections, there exists obvious insulation degradation zone, which may originate from regional humidity increase [51] or significant decrease of insulation performance of individual rail fastener [52] and track slab systems [53].

### 4.4 Experimental verification

**Fig 13:**
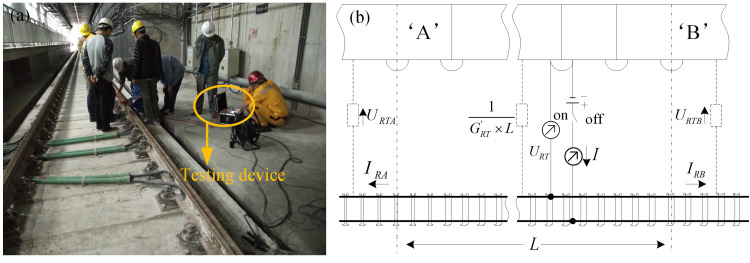
Field test of rail-to-earth conductance. (a) Rail-to-earth conductance measurement in the subway tunnel. (b) Measurement method.

Rail-to-earth conductance *G* near the monitoring site characterizes the insulation performance of rail fasteners. Due to the complex current conduction paths within the subway system, there is currently no accurate theoretical calculation method that considers the direction and amplitude of stray current leakage in three-dimensional space structures. However, since the only path for stray current to flow from the traction system into the shield tunnel and the earth environment is the rail fasteners, this paper proposes to use the rail-to-ground transition resistance along-line testing to briefly verify the accuracy of the stray current leakage location method proposed in this paper, that is, the location with lower rail-to-ground transition resistance can be considered as the area with more stray current leakage. It has been mathematically and experimentally demonstrated that the amplitude of stray current tends to increase with the increase of rail-to-earth conductance [54,55]. Thus, it could be used to reflect the degree of stray current leakage. Fig 13a presents the field tests of rail-to-earth conductance during the suspension of subway system at night. Rail-to-earth conductance in the nearby region of the monitoring site over the entire subway line is measured on Nov. 2021 and Dec. 2021 based on the method provided by EN 50122−2: 2010 [56] to evaluate the clustering results, as shown in Fig 13b. The current with an amplitude of 100 A was injected into the circuit shown in Fig 13b to obtain *U*_*RT*_, *U*_*RTA*_ and *U*_*RTB*_ and then to calculate rail-to-earth conductance *G*. The conductance *G* near each monitoring site is repeatedly tested for five times and the average value is further calculated to eliminate the interference of errors. In order to avoid the impact on daily operations of subway line, the rail-to-earth conductance test was all carried out during the night shutdown. It should be noted that, due to long distance of the entire subway line and limitation of manual test, the rail-to-earth conductance test was completed within several days.

**Fig 14:**
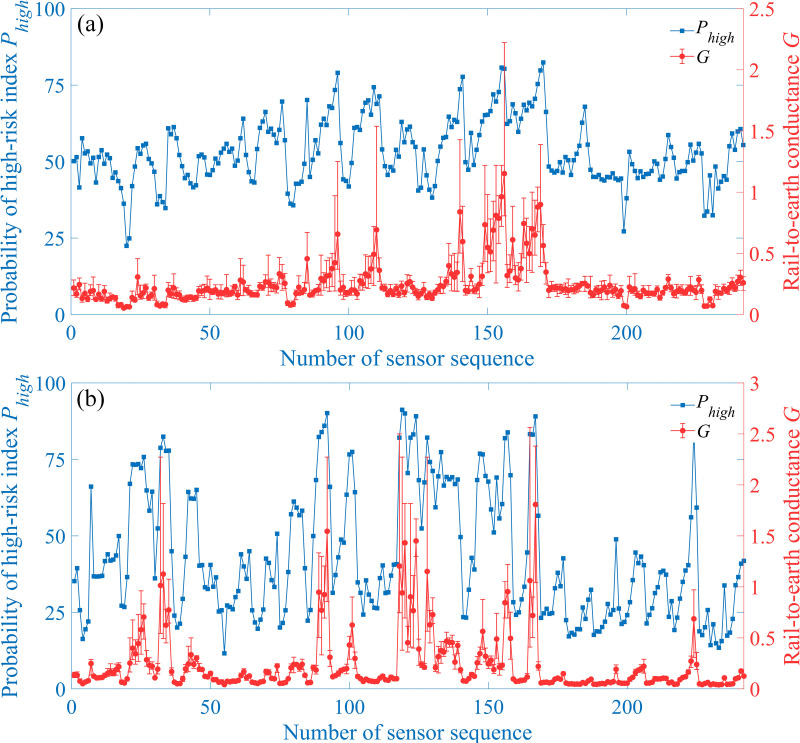
Correlation analysis between *P*_high_ and *G.* (a) Nov. 2021. (b) Dec. 2021.

Based on testing results of rail-to-earth conductance over the entire subway line, the relationship between measured rail-to-earth conductance *G* and the probability of high-risk index *P*_high_ is shown in Fig 14 along with the error bars of measured rail-to-earth conductance *G*. It can be seen that there is a strong positive correlation between the probability of high-risk index and rail-to-earth conductance. When the rail-to-earth conductance increases, the probability of high-risk index also increases accodingly. The biggest average rail-to-earth conductance in Fig 14a and b is 1.15 S/km and 1.19 S/km, corresponding to the probability *P*_high_ 80.31% and 89.06%, respectively. The smallest average rail-to-earth conductance in Fig 14a and b is 0.0533 S/km and 0.0397 S/km, corresponding to the probability *P*_high_ 36.23% and 13.49%, respectively. The correlation coefficients *R*^2^ between *P*_high_ and mean rail-to-earth conductance are also calculated based on Pearson, Spearman and Kendall methods, which are all listed in Table 5. Except for Kendall coefficient of rail-to-earth conductance measured in Nov. 2021, remaining coefficients are all above 0.75. By integrating Fig 14 and calculated *R*^2^ in Table 5, it could be concluded that the proposed method is able to effectively locate the zone where the insulation performance of rail fasteners decreases within multiple power supply sections in the subway line according to the distribution of probability of high-risk index *P*_high._

**Table 5:** Parameters of LWQPSO-SOM algorithm.

Experimental period	Pearson *R*^2^	Spearman *R*^2^	Kendall *R*^2^
Nov. 2021	0.7855	0.7957	0.6208
Dec. 2021	0.8260	0.9812	0.8829

It needs to mention that, although rail-to-earth conductance test could characterize whether there’s insulation degradation in a given zone, automated detection has not yet been implemented in this kind of measurement. It is inefficient, lagging and time-consuming to rely only on this measurement to locate the insulation degradation zone. Therefore, the proposed method realized stray current leakage location through effective combination of data obtained by the existing monitoring system (potential shift of RCS and rail potential) in the subway line and intelligent learning algorithm. There’s no need for additional new monitoring device. And therefore, there will be no interference on the daily operation of the DC rail transit system, which provides potential application prospect in the engineering. Besides, in this work, the proposed method is trained and validated based on the monitoring data of a single subway line.

### 4.5 General discussion

In this work, an AI-assisted method for structural health monitoring of subway shield tunnel was proposed using existing building structure monitoring data. In addition to damage caused by unconventional loads, stray current corrosion is the most important factor to consider in subway shield tunnels. Since insulation damage location can effectively determine the specific location of stray current leakage, insulation damage location can reflect the structural health of the current shield tunnel. This work proposes an innovative approach for locating stray current leakage based on the clustering risk index. According to the different risk of stray current leakage, the authors divided different index levels and implemented them in the form of vectors in the LWPSO-SOM algorithm. Hence, areas with higher risk are considered stray current leakage areas. Although theoretical methods have been proposed to determine the leakage points of stray current in recent years [57], this work realizes insulation degradation location based on field data, and the location results can be further applied to the daily operation and maintenance of engineering structures, showing the actual engineering value of location work. Under the influence of extreme environment, such as temperature change and high humidity environment, these factors will cause the stray current leakage amplitude to change, so that the used monitoring half-cell potential data will change. In addition, the variation of algorithm parameters will also cause the robustness of the algorithm to fluctuate. In the subsequent study, the author will further explore the influence of the algorithm parameters on the stray current leakage location accuracy.

It is also well known that polarization potential monitoring is present in every built subway tunnel structure to achieve a brief estimate of the amount of stray current leakage [58]. The proposed method effectively employs the existing large-scale data to realize the insulation deterioration location of multiple intervals of the entire metro line, and avoid secondary damage to the original engineering structure, which is important for the future application of this method in existing and new subway tunnels. In addition to this, it needs to be noted that, although the database and practical verification in this work are from the same shield tunnel, the proposed method is general and can be applied to other subway tunnel structures rather than a case study. The proposed learning-based model is trained and tested based on the monitoring database collected from a metro line. Under the influence of different influencing factors, such as variations in materials, difference in environmental conditions, AC/DC power system, etc., these mapping relationships can be captured by the machine learning model through iterative training, so that the input-output relationship under various influencing factors can be constructed. The basic correlative mechanism between stray current leakage and potential shift of RCS is approximate for each metro line. Meanwhile, almost all metro lines are equipped with measuring devices or monitoring devices for potential shift of RCS, which can realize the application of the proposed method in other metro lines. Therefore, the proposed model is general for different metro lines. As the authors mentioned in the manuscript, the collected database for model training is based on the existing monitoring system in the metro line shown in Fig 4. The proposed model is expected to be integrated into infrastructure maintenance process by connecting to the existing metro management control system, since the data can be directly obtained from the monitoring system of half-cell potential of shield tunnel.

The evolution of insulation degradation over the years, is also a crucial aspect for preventive maintenance, since there are more and more cases of subway lines interlacing in urban areas [59,60]. For example, there are works that offer a specific approach for the characterization of defects in composite materials, which could be adapted for the direct detection of insulation degradation in subway tunnels [61,62]. The reliability of our method can be further improved by combining it with a method for directly detecting insulation degradation in subway tunnels.

In the future work, given the limitations of the proposed method, the structural health monitoring based on insulation deterioration localization will focus on the impact of regional diffusion of multi-line stray currents. As scholars understand more clearly the mechanism of stray current diffusion in reinforced concrete structures [63,64], stray currents are found to not only attack the area where the shield tunnel is located, but also other buried infrastructures within the interference scope. Obvious coupling effects of stray currents are generated from different shield tunnel structures. We will further expand the complexity of the training set, such as considering the interaction and coupling of multiple subway lines, and verify the scalability of the model. In additions to this, the proposed model will be considered to be validated under extreme weather conditions, such as high humidity, temperature fluctuations, etc.

## 5 Conclusions and future works

In this work, a probability-based method for locating insulation degradation zone is proposed by integrating designed LWQPSO-SOM clustering algorithm and large-scale existing sensing data of subway line. The proposed approach is proved to be effective in locating insulation degradation zone within power supply sections through identification of the probability of high-risk index *P*_high_ defined in this work. Clustering results are validated through the field test of rail-to-earth conductance within multiple power supply sections along the subway line.

Facing with huge and complicated sensing data, LWQPSO-SOM algorithm is proposed in this work to conduct unsupervised high-performance clustering for risk level of stray current leakage. The superiority of LWQPSO-SOM in this cluster task is demonstrated by comparing results obtained by QPSO-SOM and WQPSO-SOM algorithm. 15 risk levels are classified and an evaluation parameter: probability of high-risk index *P*_high_ is calculated for stray current leakage location.

Experimental results show that, there’s a strong correlation relationship between rail-to-earth conductance *G* and the probability of high-risk index *P*_high_. Hence, it’s possible to identify insulation degradation zone according to classified indexes based on hybrid LWQPSO-SOM algorithm. In this work, the location task was conducted by employing the sensing data collected from single subway line.

Since there exists synergistic effects of stray current leakage within multiple subway lines, future work will concentrate on regional identification based on multi-line sensing data. Regional insulation degradation location model will be developed to enable simultaneous monitoring of subway tunnel structures of multiple metro lines. In this process, multi-source data fusion will be studied in the location model construction to improve the speed of data processing.
